# Efficacy of Single-Position Oblique Lateral Interbody Fusion Combined With Percutaneous Pedicle Screw Fixation in Treating Degenerative Lumbar Spondylolisthesis: A Cohort Study

**DOI:** 10.3389/fneur.2022.856022

**Published:** 2022-06-15

**Authors:** Peng Cheng, Xiao-bo Zhang, Qi-ming Zhao, Hai-hong Zhang

**Affiliations:** ^1^Department of Spine Surgery, Lanzhou University Second Hospital, Lanzhou, China; ^2^Key Laboratory of Bone and Joint Disease Research of Gansu Province, Lanzhou, China; ^3^Department of Spine Surgery, Honghui Hospital, Xi'an Jiaotong University, Xi'an, China; ^4^Department of Cardiac Surgery, Lanzhou University Second Hospital, Lanzhou, China

**Keywords:** degenerative lumbar spondylolisthesis, oblique lumbar interbody fusion, single-position, minimally invasive spinal fusion, surgical technique

## Abstract

**Objective:**

To investigate the surgical outcomes of single-position oblique lateral interbody fusion (OLIF) combined with percutaneous pedicle screw fixation (PPSF) in treating degenerative lumbar spondylolisthesis (DLS).

**Methods:**

We retrospectively analyzed 85 patients with DLS who met the inclusion criteria from April 2018 to December 2020. According to the need to change their position during the operation, the patients were divided into a single-position OLIF group (27 patients) and a conventional OLIF group (58 patients). The operation time, intraoperative blood loss, hospitalization days, instrumentation accuracy and complication rates were compared between the two groups. The visual analog scale (VAS) and Oswestry Disability Index (ODI) were used to evaluate the clinical efficacy. The surgical segment's intervertebral space height (IDH) and lumbar lordosis (LL) angle were used to evaluate the imaging effect.

**Results:**

The hospital stay, pedicle screws placement accuracy, and complication incidence were similar between the two groups (*P* > 0.05). The operation time and intraoperative blood loss in the single-position OLIF group were less than those in the conventional OLIF group (*P* < 0.05). The postoperative VAS, ODI, IDH and LL values were significantly improved (*P* < 0.05), but there was no significant difference between the two groups (*P* > 0.05).

**Conclusions:**

Compared with conventional OLIF, single-position OLIF combined with PPSF is also safe and effective, and it has the advantages of a shorter operation time and less intraoperative blood loss.

## Introduction

Degenerative lumbar spondylolysis (DLS) refers to the displacement of the lumbar vertebral body relative to the lower vertebral body due to lumbar degenerative changes. The prevalence rate is approximately 4.1 ~ 11.1% (1). When severe neurological deficits occur or medical treatment is unsuccessful, surgery can be proposed. Traditional surgical methods include anterior lumbar interbody fusion (ALIF), lateral lumbar interbody fusion (LLIF), posterior lumbar interbody fusion (PLIF), translaminar lumbar interbody fusion (TLIF), etc. However, the fixed fusion segment is long and the trauma is large. It is necessary to strip the paraspinal muscles and remove the lamina or articular process, which can lead to many postoperative complications such as ischemic contracture of the paraspinal muscles, denervation, nerve damage, and large blood vessel damage.

Therefore, orthopedic surgeons need new surgical techniques to treat DLS, such as extreme lateral lumbar interbody fusion (XLIF) and oblique lumbar interbody fusion (OLIF), which avoid the need for paraspinal muscles, articular processes, spinal canal, dural sac, etc. The destruction of the structure has the advantages of less trauma and high fusion rate. XLIF is a retroperitoneal intermuscular muscle fiber of the psoas muscle. The deficiency of XLIF is mainly reflected in the lumbar plexus branches running in the psoas muscle, especially the L4/5 segment. Thigh pain, numbness, and weakness may occur after surgery. Since there are blood vessels in the psoas major muscle vertically distributed on the lateral side of the intervertebral disc, the puncture and expansion process may damage the blood vessels, resulting in hemorrhage and hematoma. The OLIF surgical technique, which has emerged in recent years, uses an approach that targets the region between the abdominal aorta and the psoas major in the retroperitoneal space, reducing the risk of damage to the psoas major and vascular nerves without the need for neuromonitoring during surgery. OLIF has the advantages of reduced trauma, better biomechanical stability and faster functional recovery, and it is gradually being used in clinical practice (2, 3).

Percutaneous pedicle screw fixation (PPSF) is a technique of inserting pedicle screws under X-ray and is a minimally invasive treatment technique. PPSF does not require a traditional large posterior incision, extensive dissection and traction on the paraspinal muscles, which can significantly reduce postoperative pain and recovery time (4). In recent years, PPSF has been widely used in clinical surgery with satisfactory results in the treatment of spinal degenerative diseases and fractures (5, 6).

However, conventional OLIF requires intervertebral fusion cage placement in the lateral position and pedicle screw placement in the prone position, which significantly increases the operative duration and risks of life-threatening complications, such as tracheal intubation falling off during anesthesia (7). Therefore, single-position OLIF combined with PPSF may be an effective and improved treatment for DLS. This study evaluated and analyzed the clinical, surgical and radiographic outcomes of single-position OLIF combined with PPSF in treating DLS.

## Materials and Methods

### General Patient Data

**Figure 1:**
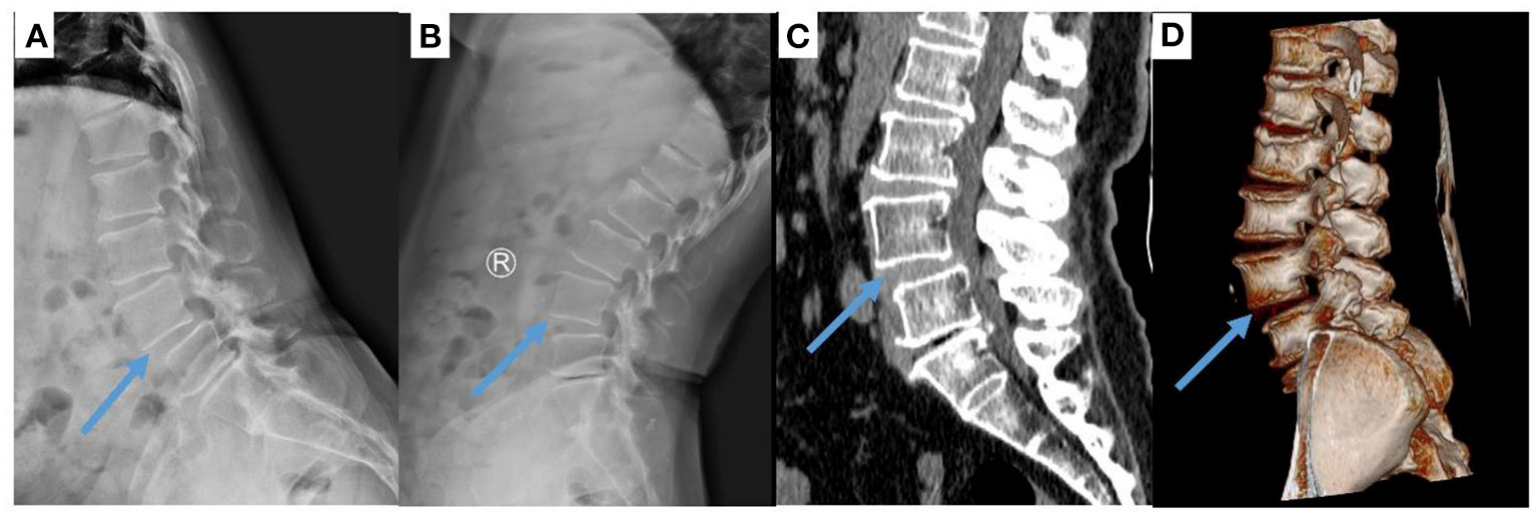
Image of L4/5 spondylolisthesis in a 65-year-old woman. **(A,B)** X-ray and **(C,D)** CT images show that the L4/5 vertebral body has slipped forward.

This study was approved by the ethics committee of the Lanzhou University Second Hospital, and this study has been reported in line with the Strengthening the Reporting of Cohort Studies in Surgery (STROCSS) criteria (8). The inclusion criteria were as follows: persistent low back pain and lower extremity pain that was unresponsive to conservative treatment; determination of the affected level by computed tomography (CT) and magnetic resonance imaging (MRI); Meyerding classification of first- or second-degree DLS (Figure 1); complete clinical and imaging data; and follow-up of more than 12 months. The exclusion criteria were as follows: history of lumbar spine surgery; patients with lumbar trauma, infection, tumor or basic disease who could nottolerate surgery; coagulation dysfunction; inability to self-evaluate due to mental illness; and missing follow-up data. There were 27 patients in the single-position OLIF group, 10 males and 17 females, with an average age of 57.70 ± 7.20 years (range 46–74), and this group included 6 cases in L3/4 segments and 21 cases in L4/5 segments. The follow-up time was 25.15 ± 4.78 (range 16–33) months. There were 58 patients in the conventional OLIF group, 24 males and 34 females, with an average age of 60.88 ± 9.51 years (range 26–75), and this group included two cases in L2/3 segments, 10 cases in L3/4 segments and 46 cases in L4/5 segments. The follow-up time was 25.91 ± 5.26 (range 14–33) months.

**Figure 2:**
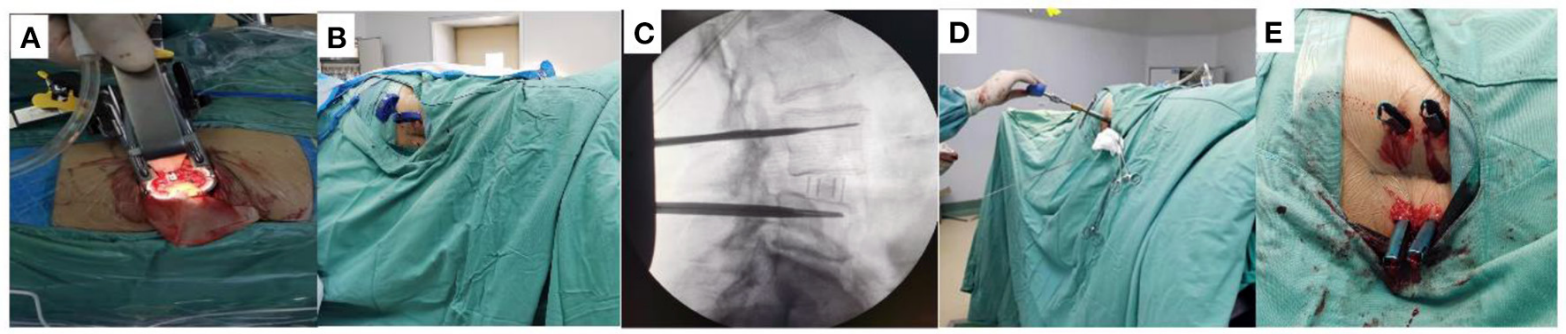
Surgical procedure for single-position OLIF. **(A)** Establish OLIF channel and place cage. **(B)** Place puncture needle in the lateral position by “freehand”. **(C)** Verification of whether the guidewire positioning is satisfactory by fluoroscopy. **(D)** Pedicle screw placement. **(E)** Installation of the pedicle screw system.

**Figure 3:**
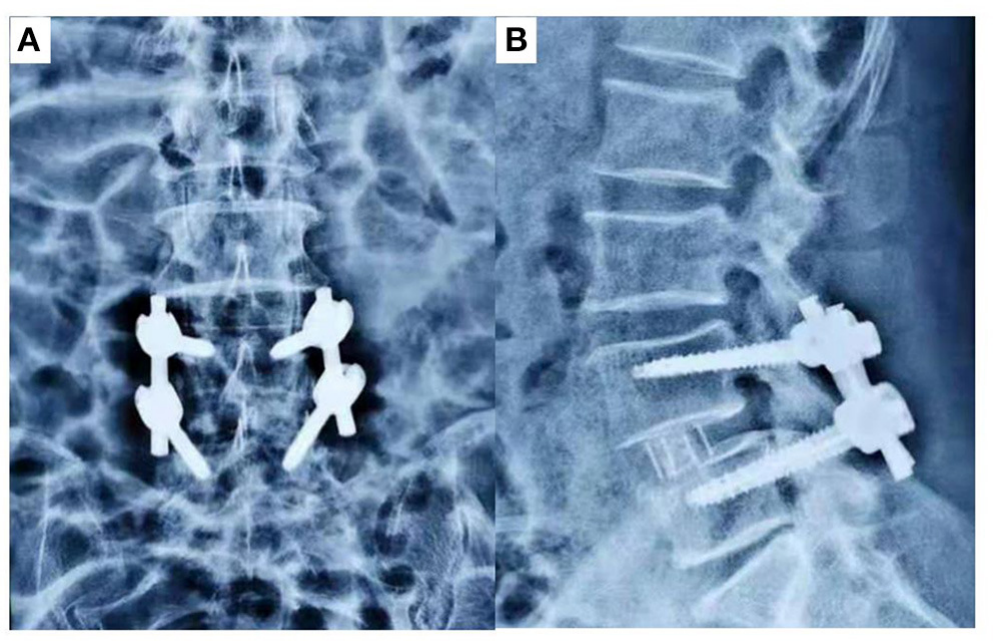
**(A)** Anterior and **(B)** lateral radiographs of a patient at 3 months postoperatively.

All the surgical procedures were performed by one orthopedic surgeon with extensive experience in spinal surgery in the Lanzhou University Second Hospital. The patient's sex and age, nail accuracy, operative duration, blood loss, hospitalization duration, complication incidence and other general data were collected and recorded. All patients were placed PPSF with “freehand,” since the OLIF procedure is already a standard procedure, not repeated in this paper (9) (Figure 2). The difference between the established and performed procedures was that we placed the cage and pedicle screws when the patient was in the right decubitus position. Since there was no difference in postoperative X-ray examination between the two groups, we reported only one case of DLS treated with OLIF (Figure 3).

### Efficacy Evaluation Indicators

Data were collected from all patients before the operation, 1-week after the operation and at the last follow-up (> 12 months). The VAS score (10) and ODI (11) were used to evaluate pain and spinal function improvements. According to Lee et al. (12), the screw placement's accuracy was assessed as follows: level 0, the screw was located entirely in the pedicle; level I, <25% of the screw diameter broke through the pedicle; level, 25–50% of the screw broke through the pedicle; and level III, more than 50% of the screw diameter broke through the pedicle. The number of screws with a placement level of 0 was recorded. The lumbar lordosis (LL) angle and intervertebral disc height (IDH) were measured by lateral X-ray. The IDH was the distance from the highest portion of the lower endplate of the cephalad vertebra to the closest portion of the upper endplate of the caudal vertebra (13).

## Statistical Analysis

The data were analyzed using SPSS (version 26.0). The results are expressed as the mean ± standard deviation. The parametric data were tested by *t*-test, and the nonparametric data were tested by chi-square test. *P* < 0.05 was considered to indicate a significant difference.

## Results

All 85 patients completed the operation. The average operative durations of the single-position OLIF group and the conventional OLIF group were 118.56 ± 15.74 min and 133.19 ± 24.94 min, respectively, and the intraoperative blood loss was 66.96 ± 14.77 ml and 88.10 ± 16.25 ml, respectively; these values of the single-position OLIF group were significantly lower than those of the conventional OLIF group (*p* < 0.05). The patients' ages in the single-position OLIF group and the conventional OLIF group were 57.70 ± 7.20 and 60.88 ± 9.51, respectively, and the hospitalization days were 7.44 ± 2.01 days and 7.34 ± 1.48 days, respectively, and the values were not significantly different (Table 1).

**Table 1:** General patient data.

**Groups**	**Single-position OLIF**	**Conventional** **OLIF**	***t***	***P***
Age	57.70 ± 7.20	60.88 ± 9.51	−1.539	0.128
Operation time	118.56 ± 15.74	133.19 ± 24.94	−2.796	**0.006**
Intraoperative blood loss	66.96 ± 14.77	88.10 ± 16.25	−5.947	**<0.001**
Hospitalization days	7.44 ± 2.01	7.34 ± 1.48	0.259	0.798

*The statistical method used is independent student t-test. When the p value is less than 0.05, the difference is statistically significant, which is displayed in bold.

### Clinical Efficacy

In the single-position OLIF group, the postoperative lumbar VAS score decreased from 6.89 ± 1.21 points to 2.85 ± 0.99 points and was 1.67 ± 0.92 points at the last follow-up. The postoperative lower extremity VAS score decreased significantly from 6.61 ± 0.98 points to 2.81 ± 0.79 points and was 1.56 ± 0.75 points at the last follow-up. The postoperative ODI decreased from 56.15 ± 8.99 to 24.30 ± 7.03 and was 16.74 ± 5.65 at the last follow-up. Similarly, in the conventional OLIF group, the postoperative VAS score significantly decreased from 7.01 ± 1.18 to 2.93 ± 1.09 and was 1.55 ± 0.78 points at the last follow-up. The postoperative lower extremity VAS score also significantly decreased from 6.57 ± 1.01 to 2.88 ± 0.94 and was 1.71 ± 0.75 at the last follow-up. The postoperative ODI decreased from 55.26 ± 7.55 to 25.72 ± 8.42 and was 16.66 ± 5.06 at the last follow-up. Compared with the preoperative VAS and ODI scores, the postoperative VAS and ODI scores in both groups were significantly improved (*P* < 0.05). There was no significant difference in VAS or ODI between the two groups preoperatively, postoperatively or at the last follow-up (*P* > 0.05) (Table 2).

**Table 2:** Comparison of the lumbar VAS score and lower limb VAS score between the two groups preoperatively, postoperatively and at the last follow-up.

**Time**	**Single-position OLIF**	**Conventional** ** OLIF**	***t***	***P***
Preoperative lumbar VAS	6.89 ± 1.21	7.01 ± 1.18	−0.475	0.636
Postoperative lumbar VAS	2.85 ± 0.99	2.93 ± 1.09	−0.321	0.749
Last follow–up lumbar VAS	1.67 ± 0.92	1.55 ± 0.78	0.599	0.551
Preoperative lower extremity VAS	6.61 ± 0.98	6.57 ± 1.01	−0.219	0.827
Postoperative lower extremity VAS	2.81 ± 0.79	2.88 ± 0.94	−0.310	0.757
Last follow-up lower extremity VAS	1.56 ± 0.75	1.71 ± 0.75	−0.865	0.391
Preoperative ODI	56.15 ± 8.99	55.26 ± 7.55	0.475	0.636
Postoperative ODI	24.30 ± 7.03	25.72 ± 8.42	−0.765	0.447
Last follow-up ODI	16.74 ± 5.65	16.66 ± 5.06	0.067	0.947

*The statistical method used is independent student t-test.

### Imaging Results

In the single-position OLIF group, the IDH was 8.30 ± 1.00 mm preoperatively, 13.58 ± 1.47 at 1 week postoperatively, and 13.31 ± 1.57 mm at the last follow-up. The LL angle was 35.31 ± 8.24 preoperatively, 47.37 ± 10.07 at 1 week postoperatively, and 46.76 ± 10.13 at the last follow-up. Similarly, in the conventional OLIF group, the IDH was 8.43 ± 1.44 mm preoperatively, 13.15 ± 1.50 mm at 1 week postoperatively, and 12.78 ± 1.64 mm at the last follow-up. The LL was 36.63 ± 8.73 preoperatively, 48.12 ± 10.39 at 1 week postoperatively, and 47.07 ± 10.09 at the last follow-up. The postoperative LL and IDH of the two groups were significantly improved compared with the preoperative LL and IDH of the two groups (*P* < 0.05), but there was no significant difference between the two groups (*P* > 0.05) (Table 3).

**Table 3:** Comparison of the ODI and IDH between the two groups preoperatively, postoperatively and at the last follow-up.

**Time**	**Single-position OLIF**	**Conventional** **OLIF**	***t***	***P***
Preoperative IDH	8.30 ± 1.00	8.43 ± 1.44	−0.424	0.673
Postoperative IDH	13.58 ± 1.47	13.15 ± 1.50	1.241	0.218
Last follow-up IDH	13.31 ± 1.57	12.78 ± 1.64	1.444	0.155
Preoperative LL	35.31 ± 8.24	36.63 ± 8.73	0.659	0.511
Postoperative LL	47.37 ± 10.07	48.12 ± 10.39	−0.312	0.755
Last follow-up LL	46.76 ± 10.13	47.07 ± 10.09	−0.130	0.897

*The statistical method used is independent student t-test.

### Complications

In the single-position OLIF group, the incidence of complications was 21.43% (6/27); three patients had transient high pain or numbness, and the symptoms disappeared naturally within half a year. One patient had segmental vascular injury. One patient had pain in the operation area of the iliac bone. One patient had cerebrospinal fluid leakage. After lying on his back and rehydration for 3 days, the symptoms of dizziness gradually relieved. One patient developed an abdominal bulge at the wound site. In the conservative OLIF group, the incidence of complications was 27.59% (16/58). During the operation, there were two cases of peritoneal injury and two cases of endplate injury. Five patients had transient thigh pain or numbness, one patient had low back pain, and two patients had lumbar myasthenia, and the symptoms gradually disappeared within half a year without treatment; two patients had transient hip flexion weakness, and the symptoms disappeared after nerve detumescence treatment; two patients had cerebrospinal fluid leakage, and the symptoms of dizziness gradually decreased after lying down and rehydration for seven days. There was no significant difference in the incidence of complications between the two groups (χ2 = 0.276, *P* = 0.599). In the single-position OLIF group, the pedicle screws placement accuracy was 96.30% (104/108), and that in the conventional OLIF group was 94.83% (220/232). There was no significant difference between the two groups (χ2 = 0.254, *P* = 0.614).

## Discussion

Over time, doctors have increasingly favored minimally invasive surgery. While ensuring clinical results, minimally invasive procedures minimize surgical trauma and ease patient suffering. Various minimally invasive techniques have been applied in the clinic and have benefitted many patients (14, 15). In recent years, the clinic has widely used minimally invasive oblique lateral spinal fusion surgery via OLIF. This procedure uses extraperitoneal blood vessels and the psoas muscle space for entry, allows the implantation of a larger fusion cage, increases the bone contact surface, and thus increases the fusion rate. This approach also allows complete opening of the intervertebral space, expansion of the intervertebral foramen area, and restoration of the spinal canal volume to achieve indirect decompression (2, 16). OLIF is widely used because of its ability to provide indirect decompression and its minimal invasiveness; it also helps restore the sagittal curve and coronal balance. It can be applied to treat various lumbar degenerative diseases, especially lumbar spondylolisthesis (17). Because of the lack of an ideal effect and the insufficient rotational stability achieved with a fusion cage alone, bilateral pedicle screw fixation is still the gold standard (18).

However, in conventional OLIF, the patient usually needs to be in the lateral position for cage placement and then transitioned to the prone position for pedicle screw placement. In this study, single-position OLIF significantly reduced the intraoperative blood loss and shortened the operation time compared with conventional OLIF. Single-position OLIF does not require repositioning, which helps save operative time. Additionally, because the operation time is reduced, the intraoperative blood loss and anesthesia time are reduced, which helps decrease the risk of infection and anesthesia decannulation. Although no patients with anesthesia decannulation and infection were observed in this study, patients may benefit from the close attention of anesthesiologists and strict compliance with routine aseptic procedures.

Similar to previous reports, the advantages of single-position OLIF and single-position LLIF over dual-position appear to be consistent and reduce the occupancy time of the operating room and workforce requirements (19, 20). The LLIF approach is an excellent choice for sagittal and coronal deformity correction, especially for lumbar degenerative scoliosis with lateral slippage (21). However, the LLIF approach may not be suitable for severe central canal stenosis, bony lateral recess stenosis, and high-grade slippage (22). Additionally, it is not suitable for patients with a history of retroperitoneal surgery or a retroperitoneal abscess, as well as patients with abnormal vascular anatomy. Potential risks of this technique include lumbar plexus, psoas, and bowel injury, especially at the L4/5 level (23). Vascular injury can be difficult to control once it occurs and represents another risk of the lateral transpsoas approach (21, 24). The shortcomings of OLIF are mainly reflected in the limitation of the approach and the limitation of the operative segment (only used for L2-5), the limitation of indirect decompression, and it cannot directly remove the prominent disc. In conclusion, single position technology may be an effective improvement measure.

In addition, the VAS score and ODI in this study were significantly lower after the operation than before the operation. The lumbar and lower limb symptoms were significantly improved. Similarly, IDH and LL on imaging were considerably enhanced compared with those before operation. Loss of the LL angle and changes in the IDH are closely related to the development of DLS. Therefore, it has important clinical significance to study the changes in the LL and IDH in DLS patients. The loss of LL is a key cause of low back pain in patients (25), and the restoration of the IDH at the affected segment improves the compression of the nerve root at the corresponding segment. In this study, single-position and conventional OLIF can significantly restore lumbar lordosis and intervertebral space height. There was no significant difference in the VAS, ODI, IDH, and LL between the two groups at the last follow-up. In short, single-position OLIF has clinical efficacy, high safety, and feasibility that are similar to those of conventional OLIF. Similar results have been obtained in other studies (9, 26).

The two groups of patients inevitably had different degrees of complications. Vascular injury, which is mainly the injury of vertebral segmental vessels and iliac vessels, is a common complication of OLIF (27). The risk of vascular injury by the OLIF technique is mainly related to the process of incision exposure, separation of psoas muscle and vascular sheath, and deep clearance of intervertebral space (28). Endplate injury mostly occurs in patients with osteoporosis (2). Improper operations during surgery, such as directly using a sharp reamer to remove the nucleus pulposus or following the wrong direction during intervertebral disc cleaning, can cause endplate damage. For patients with intraoperative endplate injury, screw fixation is necessary (29). Among our patients who underwent single-position OLIF, three patients (11.11%) had transient leg weakness during follow-up, and four patients (8.62%) in the conventional OLIF group had transient leg weakness during follow-up. The numbers were within the normal range (6.1–60.3%) (16, 30, 31). It can be seen that there is no significant difference between single-position OLIF and traditional OLIF in the incidence of postoperative complications, which is related to the OLIF procedure (32). Postoperative thigh numbness and hip flexion weakness may be caused by retraction of the psoas muscle and associated sensory nerves (33). These symptoms are mostly transient, and postoperative rehabilitation exercises can facilitate recovery in a short period. Hiyama et al. (34) reported that to prevent postoperative motor weakness regardless of the operation time, surgeons should be aware of the potential for surgical invasive of the psoas muscle during LLIF in older people ADDIN EN.CITE (34).

Pedicle screw misplacement is a common complication in spinal surgery. This study used “freehand” screw placement. “Freehand” is the manual placement of PPSF without using a robot, but still requires C-arm perspective assistance. According to reports, the displacement rate of “freehand” pedicle screw placement is 1.5–14.3% (35–37). Although the development of robotic technology (computer navigation technology) has helped to improve accuracy (38, 39), sometimes hospitals do not have computer technology navigation technology and often still perform “freehand” operations.

The single-position OLIF may be a new technology worthy of recommendation. Recently, Kotani et al. (40) found that single-position OLIF can provide a comparable fusion rate, segmental radiologic alignment, and symptomatic adjacent segment degeneration to MIS-TLIF surgery. Pham et al. (41) presented a novel technical report on the recommended workflow of simultaneous robotic single-position OLIF and demonstrated the'feasibility of placement of sacroiliac fixation in the lateral decubitus position. In addition, Diaz-Aguila et al. (26) found that robot-assisted OLIF can reduce the operative time while ensuring accurate and timely screen placement with minimal complications. As medical technology continues to develop, minimally invasive, robot-assisted surgical treatment will be increasingly used. We expect to see more reports on robot-assisted treatments for lumbar degenerative disease. In short, single-position OLIF serves as a safe, minimally invasive and effective surgical modality that saves valuable operating room time and is worth popularizing.

Although OLIF has advantages over conventional surgery in terms of the operative duration. However, we believe that surgeons should pay attention to the following factors. First, surgeons must have sufficient experience with OLIF combined with “freehand” placement of PPSF. Second, the patient's position is significant. There is a learning curve required for the surgeon to insert the percutaneous pedicle screw on the patient's right side (37, 42). On the one hand, being too close to the bed will affect the fluoroscopy. On the other hand, being too far away from the bed will limit the puncture angle of PPSF. ADDIN EN.CITE (42). According to our experience, the average time required to insert each percutaneous pedicle screw on the patient's right side was significantly longer than that required to insert each screw on the left side. This difference may occur because it is easier to apply force while inserting the percutaneous pedicle screw from the left side. It will limits the surgeon's ability to place the hand low enough to medialize the pedicle screw on the underside (42). Therefore, the patient's position should be as close as possible to the side of the operating bed so as not to block the operating bed when the nail is placed on the right side. In our opinion, the ideal position is from the edge of the bed to a quarter of the width of the bed.

There are some limitations to our study. First, because the single-position OLIF method has not yet been popularized, the sample size of our study is small and the follow-up time is short, and larger sample size and longer follow-up time are needed to confirm its safety and efficacy. Second, the survey subjects were limited to LDS patients treated at Lanzhou University Second Hospital. There may be differences among medical institutions due to differences in medical equipment. Third, surgeons may have varying experiences with OLIF techniques.

## Conclusion

Single-position OLIF combined with PPSF significantly shortens the operation time, reduces the amount of intraoperative blood loss, is clinically effective, substantially improves the operation efficiency, and has good feasibility and safety. Thus, the treatment of DLS with single-position OLIF may be worth promoting.

## Data Availability Statement

The raw data supporting the conclusions of this article will be made available by the authors, without undue reservation.

## Ethics Statement

The studies involving human participants were reviewed and approved by the Ethics Committee of the Lanzhou University Second Hospital. The patients/participants provided their written informed consent to participate in this study. Written informed consent was obtained from the individual(s) for the publication of any potentially identifiable images or data included in this article.

## Author Contributions

PC and X-bZ conceived the study design and drafted the manuscript. Q-mZ supervised the data collection. H-hZ contributed to the revision. All authors contributed to the article and approved the submitted version.

## Funding

This work was supported by the Natural Science Foundation of China (no. 31960175), the Natural Science Foundation of Gansu Province (no. 21JR1RA127), and the Natural Science Foundation of Gansu Province (no. 17JR5RA235).

## Conflict of Interest

The authors declare that the research was conducted in the absence of any commercial or financial relationships that could be construed as a potential conflict of interest.

## Publisher's Note

All claims expressed in this article are solely those of the authors and do not necessarily represent those of their affiliated organizations, or those of the publisher, the editors and the reviewers. Any product that may be evaluated in this article, or claim that may be made by its manufacturer, is not guaranteed or endorsed by the publisher.

## Abbreviations

OLIF: Oblique lateral interbody fusion
PPSF: Percutaneous pedicle screw fixation
DLS: Degenerative lumbar spondylolisthesis
VAS: Visual Analog Scale
ODI: Oswestry Disability Index
IDH: Intervertebral disc height
LL: Lumbar lordosis
ALIF: Axial lumbar interbody fusion
LLIF: Lateral lumbar interbody fusion
PLIF: Posterior lumbar interbody fusion
TLIF: Translaminar lumbar interbody fusion
XLIF: Lateral lumbar interbody fusion
STROCSS: Strengthening the Reporting of Cohort Studies in Surgery
CT: Computed tomography
MRI: Magnetic resonance imaging
MIS-TLIF: Minimally invasive transforaminal interbody fusion.
